# KIF23 is an independent prognostic biomarker in glioma, transcriptionally regulated by TCF-4

**DOI:** 10.18632/oncotarget.8261

**Published:** 2016-03-22

**Authors:** Lihua Sun, Chuanbao Zhang, Zhengxiang Yang, Yiping Wu, Hongjun Wang, Zhaoshi Bao, Tao Jiang

**Affiliations:** ^1^ Department of Molecular Neuropathology, Beijing Neurosurgical Institute, Capital Medical University, Beijing, China; ^2^ Department of Neurosurgery, The Affiliated Wuxi People's Hospital of Nanjing Medical University, Wuxi, China; ^3^ Department of Neurosurgery, 2nd Affiliated hospital of Harbin Medical University, Harbin, China; ^4^ Department of Neurosurgery, Beijing Tiantan Hospital, Capital Medical University, Beijing, China; ^5^ Center of Brain Tumor, Beijing Institute for Brain Disorders, Beijing, China; ^6^ China National Clinical Research Center for Neurological Diseases, Beijing, China

**Keywords:** glioma, prognostic, KIF23, TCF-4, proliferation

## Abstract

Kinesin family member 23 (KIF23), a nuclear protein and a key regulator of cellular cytokinesis, has been found to be overexpressed as an oncogene in glioma. However, the prognostic and clinicopathological features of glioma with KIF23 expression was not clear yet. Here, we analyzed KIF23 expression pattern by using whole genome mRNA expression microarray data from Chinese Glioma Genome Atlas (CGGA) database (http://www.cgga.org.cn), and found that KIF23 overexpression was significantly associated with high grade glioma as well as the higher mortality in survival analysis (log-rank test, p<0.01). The results of the three other validation datasets showed similar findings. Furthermore, KIF23 also served as an independent prognostic biomarker in glioma patients. Finally, functional assay showed that reduction of KIF23 suppressed glioma cell proliferation both in vivo and vitro. Additionally, we found that KIF23 was regulated by TCF-4 at transcriptionally level. Therefore, this evidence indicates KIF23 over-expression is associated with glioma malignancy and conferred a worse survival time in glioma, which suggests KIF23 is a new novel prognostic biomarker with potential therapeutic implications in glioma.

## INTRODUCTION

Glioma, the most common malignant neoplasm of central nervous system, remains one of the most lethal tumors for its refractory features to conventional therapies [1]. The median survival of patients with primary glioblastoma (GBM), the most malignant and frequent type of glioma [2], is only 1 year [3, 4]. However, the survival time of GBM patients, ranging from months to over 5 years following diagnosis [5]. This phenomenon may due to the heterogeneity of tumor, indicating the limitations of the current morphology based diagnosis, prediction and prognosis evaluation. Given that the identification of biomarkers can develop patient-specific treatments and improve the clinical outcome of glioma patients [6–8], more and more molecular markers (IDH1/2, MGMT, ATRX, etc) have been identified, many of which are used for diagnosis, classification, therapy response assessment, and prognosis evaluation [9–13].

Kinesin family member 23 (KIF23) is a nuclear protein, which is essential for spindle midbody formation, has been identified as a key regulator of cytokinesis [14–17]. And reduction of KIF23 could suppress cell proliferation and xenograft growth. However, the prognostic and molecular features of glioma with KIF23 expression is unknown, and the regulation mechanism of KIF23 is still unclear. In this study, we evaluated the expression pattern, prognostic value and biological associations of KIF23 from RNA expression profilings of glioma samples, as well as from in vitro and in vivo assays. We demonstrated that TCF-4 regulated KIF23 expression at transcriptional level. These results suggest KIF23 is a novel biomarker with potential important therapeutic implications in glioma.

## RESULTS

### KIF23 expression is associated with glioma grades and shows a subtype preference

**Figure 1:**
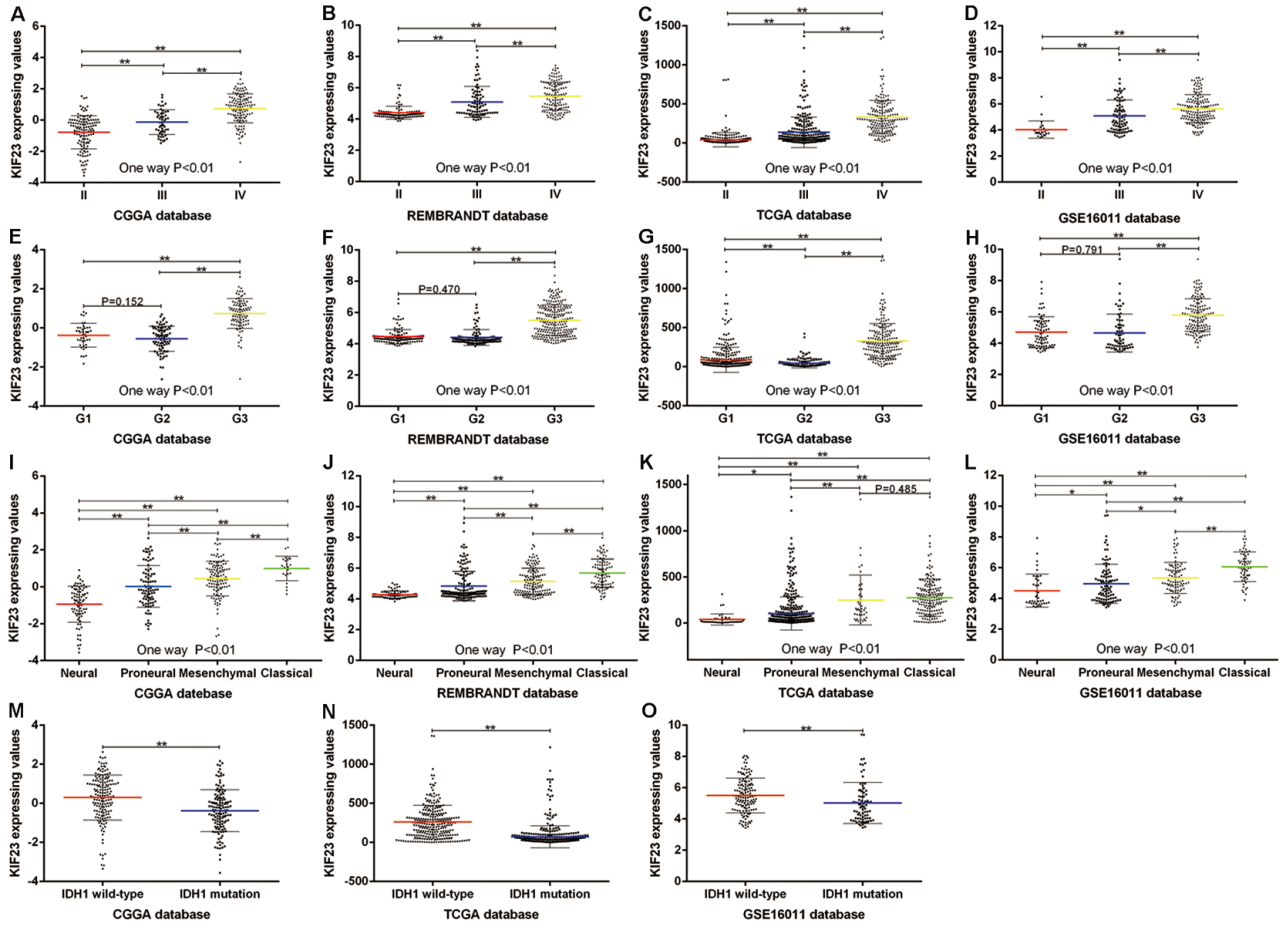
KIF23 expression pattern in CGGA and other validation datasets **A–D.** KIF23 expression is positively correlated with tumor grade. **E–H.** KIF23 expression shows G3 subtype preference according to CGGA classification system. **I–L.** KIF23 expression shows Classical subtype preference according to TCGA classification system. **M–O.** Patients with wild-type IDH1 have higher expression of KIF23 than those with mutant IDH1. A single spot is the KIF23 expression value of an individual patient. Lines in the middle are the mean expression value. Error bars represent standard deviation (SD). * P<0.05, ** P<0.01.

We screened the differentially expressed genes from the Chinese Glioma Genome Atlas (CGGA) dataset, and found that KIF23 was significantly differentially expressed among WHO II, III and IV tumor samples. KIF23 expression was positively correlated with tumor grade (P<0.01). These results were validated in TCGA, REMBRANDT and GSE16011 databases. (Figure 1A-1D). In order to analyze KIF23 expression value in the different molecular subtypes of glioma, we annotated the 4 datasets using TCGA and CGGA classification systems by Prediction Analysis of Microarrays (PAM) [18, 19]. Based on CGGA classification system, KIF23 expression was higher expressed in G3 subtype (low IDH1 mutation and 1p/19q co-deletion) by comparison with G1, G2 subtype (Figure 1E-1H). Furthermore, using TCGA subtype classification system, the Classical subtype (characterized by EGFR amplification) had the highest KIF23 expression while the Neural subtype showed the lowest KIF23 expression in CGGA, REMBRANDT and GSE16011 datasets (Figure 1I-1K). Though there's no difference between Mesenchymal subtype and Classical subtype in TCGA database, the mean value of KIF23 in Classical group still showed increasing trend compared to that of Mesenchymal subtype (Figure 1L). These results indicated that KIF23 had a G3 and Classical subtype preference. Additionally, glioma samples with wild-type IDH1 showed higher expression of KIF23 than those with mutant IDH1 (Figure 1M-1O). The correlations among KIF23, subtype, WHO grade and IDH1 mutation were also shown in Figure 3.

### KIF23 is an independent prognostic biomarker for glioma patients

**Figure 2:**
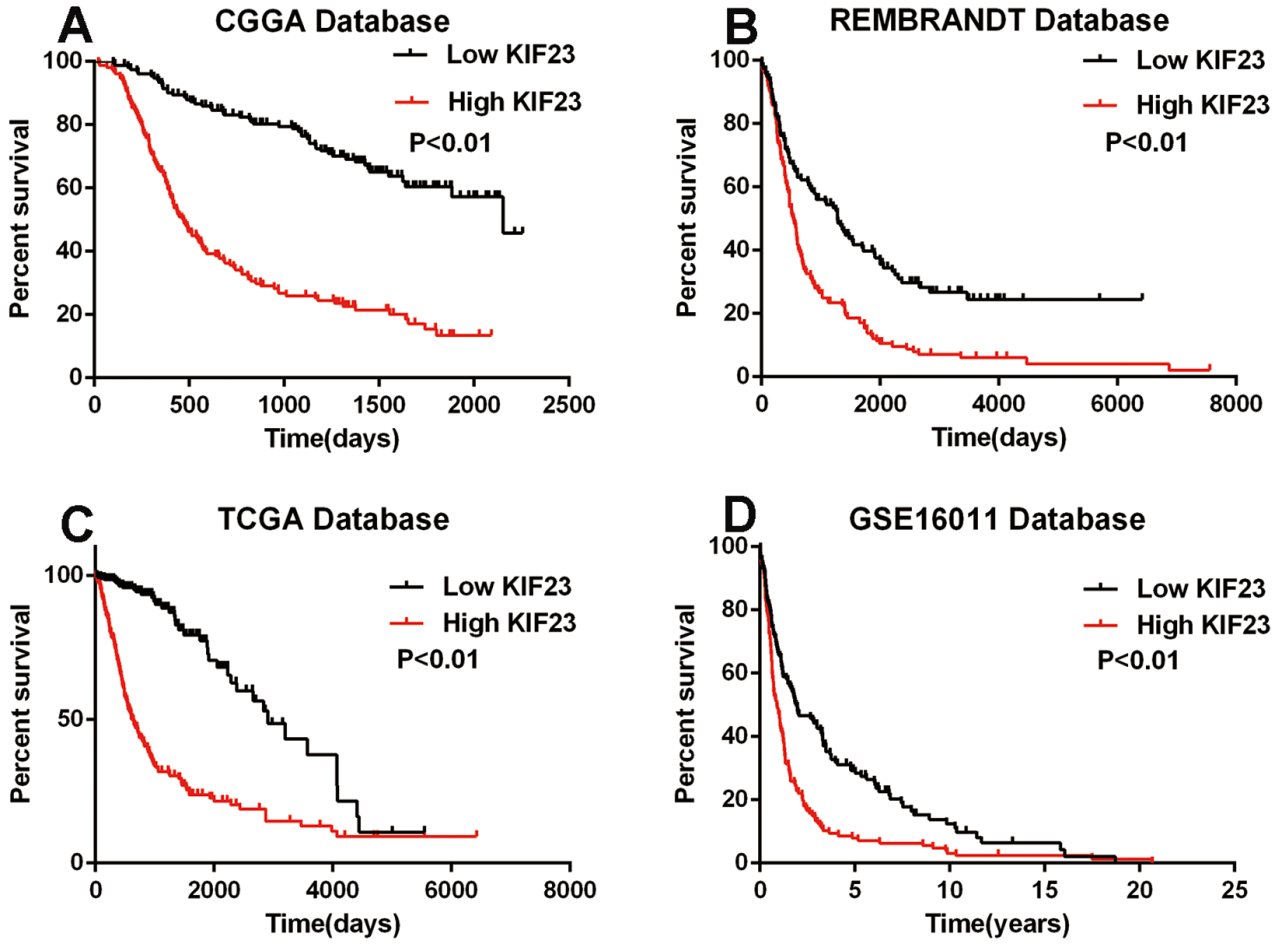
The prognostic value of KIF23 in glioma High KIF23 confers a poor survival in **A.** CGGA (median survival days of low and high group are 2153 vs 470), **B.** REMBRANDT (median survival days of low and high group are 1284 vs 532), **C.** TCGA (median survival days of low and high group are 2907 vs 607) and **D.** GSE16011 (median survival years of low and high group are 1.92 vs 0.88) datasets. High group, patients with higher KIF23 expression than the median value or equal to the median one. Low group, patients with lower KIF23 level than the median one.

The association of KIF23 expression with prognosis of glioma patients was investigated through Kaplan-Meier survival curve analysis with a log-rank test of 305 glioma patients in CGGA dataset. Patients with high KIF23 expression (median survival, 2153 days) had a significantly worse overall survival time in contrast to those with low KIF23 expression (median survival, 470 days) (P<0.01) (Figure 2A). Furthermore, Similar result was also observed in the three other validation sets (P<0.01, respectively) (Figure 2B-2D). To further determine the prognostic value of KIF23 in glioma patients, a univariate Cox regression analysis was employed. As shown in Table 1, high KIF23 expression was shown to be a risk factor for glioma patients [P<0.01, HR 4.241, 95 % confidence interval (CI) 3.037-5.923]. Additionally, some other factors including IDH1 mutation state, KPS, age, and tumor grade, were significantly associated with the overall survival of glioma patients. Next, we performed multivariate Cox regression analysis incorporating KIF23 expression, age, IDH1 mutation and tumor grade. The analysis revealed that KIF23 expression was an independent prognostic factor for the overall survival of glioma patients. Taken together, these results imply that KIF23 might be a useful independent prognostic biomarker for glioma patients.

**Table 1:** Cox proportional hazard regression analyses of KIF23 expression and clinicopathological factors affecting overall survival of glioma patients

Variable	Univariate			Multivariate		
	HR	95% CI	p Value	HR	95% CI	p Value
Gender (male vs female,)	1.272	0.932-1.737	0.130			
KIF23 (high vs low)	4.241	3.037-5.923	<0.01	2.370	1.559-3.602	<0.01
IDH1 (mutation vs wild-type)	0.372	0.267-0.518	<0.01	0.844	0.553-1.288	0.431
Grade progression	3.036	2.482-3.713	<0.01	2.408	1.851-3.133	<0.01
Increasing Age	1.041	1.027-1.056	<0.01	1.013	0.998-1.028	0.083
Increasing KPS	0.958	0.948-0.968	<0.01	0.969	0.957-0.981	<0.01

HR, hazard ratio

### KIF23 is tightly correlated with cell cycle process

**Figure 3:**
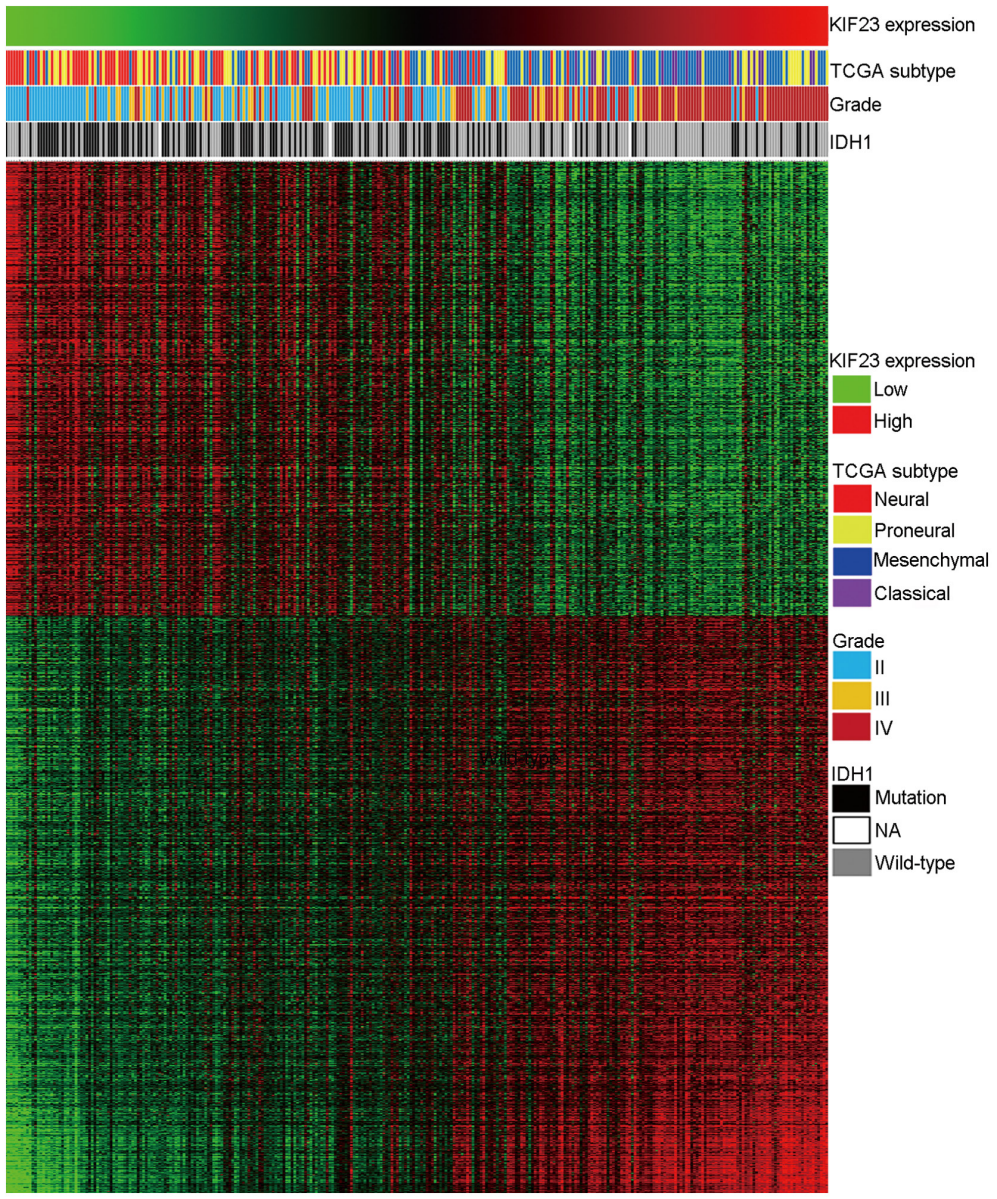
Heatmap of the KIF23 correlated gene-expression signature and KIF23 expression shows a Classical subtype, IDH1 wild-type and grade preference For each patient, TCGA subtype is annotated as previous reported and listed in the upper panel as well as the IDH1 status and tumor grade, which were obtained from CGGA database. The positively and negatively correlated genes are showed in the lower part. Columns represent patients and rows represent probe sets. Patients are arranged from left to right by increasing values of KIF23. Expression levels of the probe sets are represented by color, with green demonstrates lower and red means higher than the median value.

**Figure 4:**
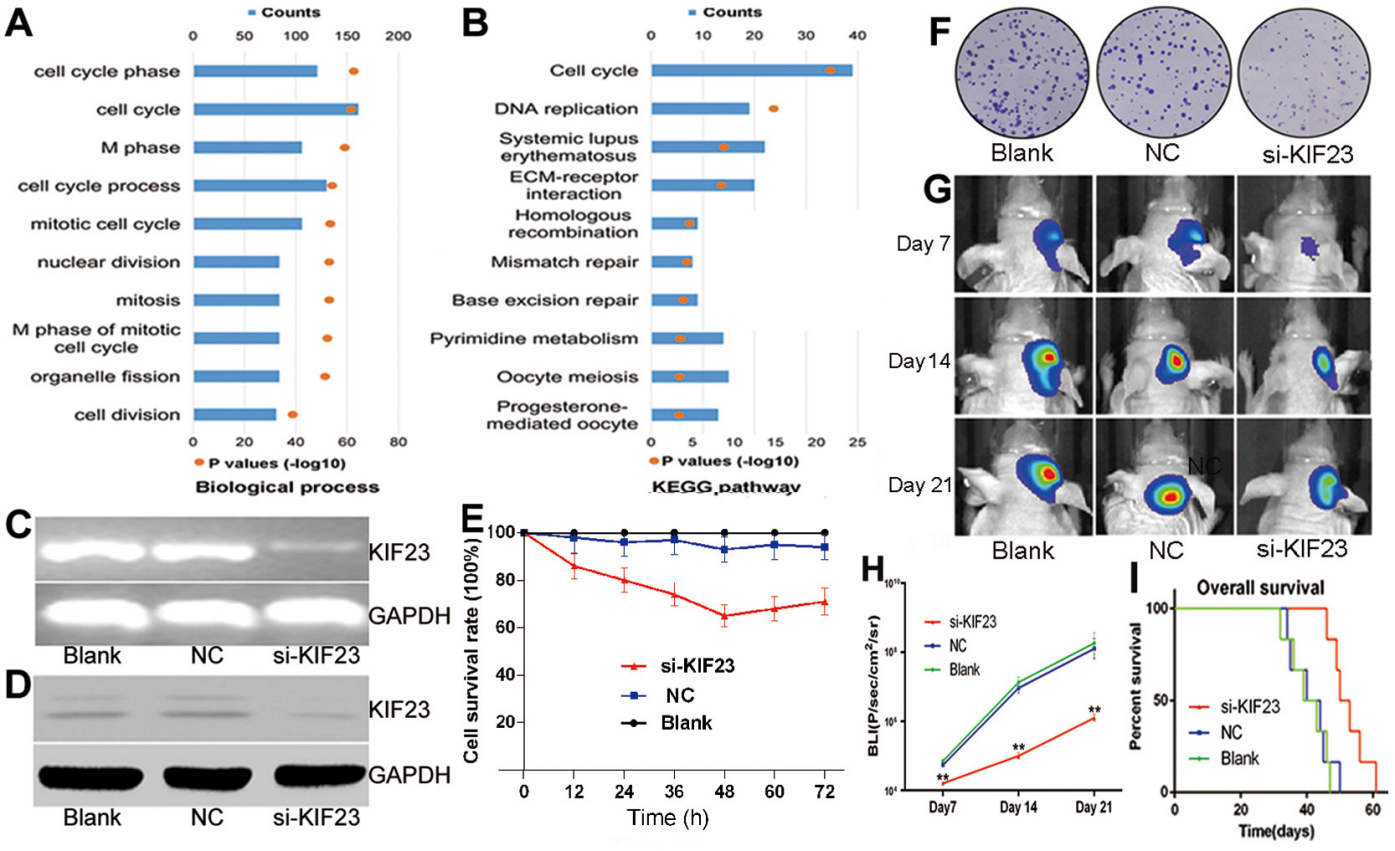
KIF23 is a tumor oncogene in glioma **A&B.** Biological processes and KEGG pathway analysis of KIF23 positively correlated genes in glioma, respectively. The upper X-axis indicates gene counts, and the lower X-axis indicates the adjusted (−log 10) p values. **C&D.** KIF23 mRNA and protein are down-regulated by KIF23 small interfering lentivirus (si-KIF23) in U87cells, respectively. **E.** MTT assay shows that U87 cell growth is significantly inhibited by si-KIF23. **F.** Clonogenic assay indicates that si-KIF23 can suppress U87MG cell clone formation. **G.** Representative images of intracranial glioma mice on days 7, 14 and 21. **H.** Plot of the Fluc activity by bioluminescence imaging for intracranial tumors. **I.** Reduction of KIF23 expression prolongs intracranial glioma mice's overall survival days. NC means negative control group, si-KIF23 means KIF23 small interfering lentivirus treatment group.

To investigate biological processes associated with KIF23 expression in glioma, Pearson correlation analysis between KIF23 expression and other genes in whole genome gene profiling were performed in 305 CGGA glioma samples. 1005 probes were positively correlated (R>0.5, P<0.01) and 798 probes were negatively correlated (R<-0.5, P<0.01) (Figure 3, Supplementary Table S1). Then, the significantly positively correlated genes were used for GO analysis. The top 10 GO terms indicated that KIF23 was significantly associated with gene sets related to cell cycle (Figure 4A, Supplementary Table S2). An analysis of the KEGG pathway mainly denoted dysfunction of cell cycle (P =2.06E-22) and DNA replication (P =1.48E-15) (Figure 4B). These analyses indicate that KIF23 might have an essential role in glioma cell proliferation.

### Reduction of KIF23 suppresses glioma cell proliferation and intracranial tumor growth

To explore the functional role of KIF23 in glioma, small interfering lentivirus was used to silence KIF23 expression. Deceased KIF23 expression were found in U87 glioma cells at both mRNA and protein levels using RT-PCR and Western blot analysis (Figure 4C&4D). Cell viability was measured by MTT and Clonogenic assays. As shown in Figure 4E&4F, antagonism of KIF23 expression suppressed the cell growth and the formation of cell clones. To further assess the effect of KIF23 activity on tumor growth in vivo, a U87 intracranial glioma mouse model treated with si-KIF23 was applied. There was a marked decrease of tumor mass between the si-KIF23 group and the control group indicated by bioluminescence imaging (Figure 4G&H). Additionally, reduction of KIF23 expression prolonged intracranial glioma mice's overall survival days by compared to the control (Figure 4I).

### KIF23 is transcriptional regulated by TCF-4

**Figure 5:**
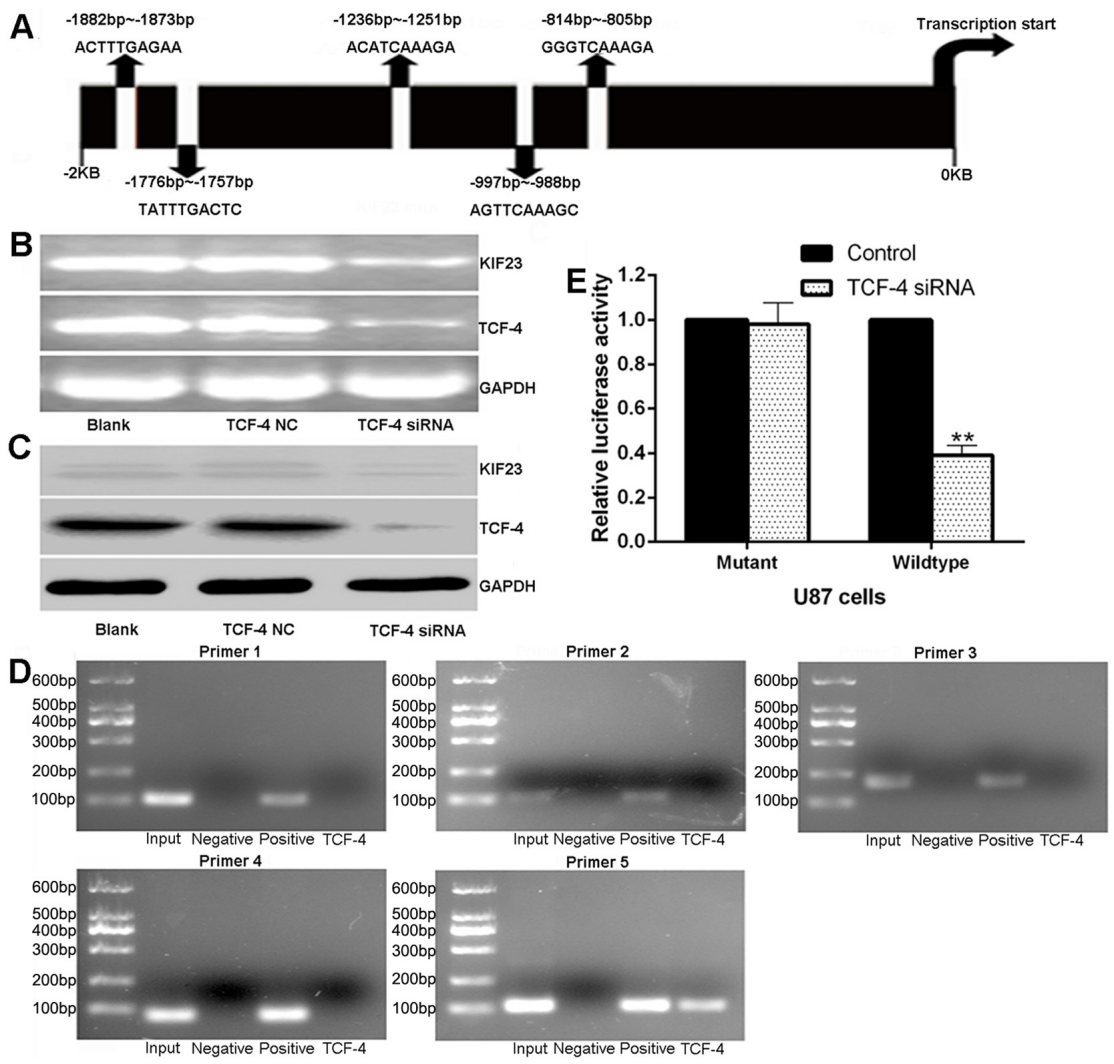
KIF23 is regulated by TCF-4 at transcriptional level **A.** Diagram of the 5 putative TCF-4 binding sites in KIF23 promoter region. **B&C.** TCF-4 siRNA significantly reduces TCF-4, KIF23 mRNA and protein expression. **D.** CHIP assay reveals that only PCR with primer 5 shows amplifications, no amplifications are detected with other primers or the negative control. **E.** Effects of TCF-4 siRNA on the activity of the KIF23 promoter in U87 cells, and mutations of the TCF binding sites can abolish the inhibitory effect of TCF-4. Error bars represent SD of triplicate independent experiments. ** means P<0.01 by contrast to the control group.

To identify the upstream regulators of KIF23, we scanned KIF23 gene promoter region for regulating DNA-binding elements in GenBank nucleotide database. Among all the factors, we focused on transcription factor TCF-4, an essential component of the Wnt/β-catenin /TCF pathway which has important role on cell proliferation [20], with 5 putative binding sites in the KIF23 promoter region, showing a high degree of homology to the core consensus sequence (CTTTGA or TCAAAG) (Figure 5A). Interestingly, we found that KIF23 expression was tightly associated with Wnt pathway activity by Gene Set Enrichment Analysis (GSEA) algorithm. GSEA is a computational method that assesses coordinate expression changes at a gene set level (Supplementary Figure S1A & S1B). To further investigate the functional interaction between TCF-4 and KIF23 expression, TCF-4 small interfering RNAs (siRNAs) were used to knockdown TCF-4 expression. Significant reductions of TCF-4 and KIF23 expression were detected (Figure 5B&C). To further determine the binding of TCF-4 to the KIF23 promoter, ChIP assays were applied in U87 cells. Five primers were designed to amplify the putative binding sites as predicted. As shown in Figure 5D, only primer 5 which targeted binding site 5 showed amplifiable product. In contrast, no amplifications were detected with other primers. To determine the specificity of the KIF23 promoter binding site 5 for transactivation by TCF-4, pGL3-mutant-KIF23-promoter, pGL3-Wildtype-KIF23-promoter luciferase plasmids for the KIF23 promoter consensus binding sites located at −814/−805 bp was constructed and transfected into U87 cells. Wild-type KIF23 promoter demonstrated a low activity after reduction of TCF-4 while mutant KIF23 promoter could abolish the inhibitory effect of TCF-4 siRNA on luciferase activity (Figure 5E). Taken together, these results support the hypothesis that TCF-4 regulates KIF23 through binding KIF23 promoter at the transcriptional level.

## DISCUSSION

The traditional classification of glioma by WHO is based on histological morphology, and the same treatments were applied to the patients according to tumor grade [21]. However, it is common that patients with the same WHO grade have different clinical features and outcome, for which may affected by mutant or changed expression of key genes [9, 10]. A better understanding of the molecular and genomic alterations in glioma genesis and progressive process may provide better diagnose, prognoses of glioma patient, as well as advances the development of personalized therapeutics to improve the clinical outcome [22]. Thus, identification of novel biomarker directed against molecular target based on large glioma samples is a new era for patient-specific therapy.

KIF23 is a nuclear protein, which localizes to the interzone of mitotic spindles and acts as a plus-end-directed motor enzyme that moves anti-parallel microtubules [17]. Later research found that in proliferation cells, KIF23 is essential for midbody formation and completion of cell cytokinesis, for which accumulates to the central antiparallel overlap zone of the microtubule-based structures and recruits various downstream cytokinesis factors to the site of division [14, 23, 24]. And mutant KIF23 without nuclear localization signals lead to cell cycle arrest, further demonstrating the function of KIF23 [15]. KIF23 was elevated in glioma tissues and cell lines compared to normal brains, and silencing KIF23 expression inhibited the growth of glioma cells both in vitro and in vivo [25]. Given that KIF23 is vital for cytokinesis and its effects on cell growth, KIF23 is a possible therapeutic target in glioma. However, only 11 glioma samples were employed in the above study, the relationship between clinical features and KIF23 expression was not investigated either. So, further analyses on large samples are urgent and valuable.

In this study, we evaluated KIF23 expression pattern in 305 glioma samples of CGGA database, the results showed that KIF23 expression was positive correlated with tumor grades. Furthermore, the highest KIF23 expression was identified in CGGA G3 group and TCGA Classical subtype. Both of the two subtypes tend to have a worse survival compared to other subtypes in the same classification database [18, 19]. Additionally, KIF23 expression was increased in wild-type IDH1 group, which has a poorer survival by contrast to mutant group [10, 26]. These results indicated that KIF23 expression is elevated in malignant groups with poor clinical outcomes. Patients with higher expression of KIF23 had a shorter survival time than those with lower KIF23 expression. Furthermore, to evaluate whether KIF23 could act as an independent prognostic biomarker for glioma patients, we performed a stepwise, multivariate Cox regression analysis incorporating KIF23 expression, KPS score, age, tumor grade and IDH1 mutation status. This analysis revealed that KIF23 expression level was an independent prognostic factor for the overall survival of glioma patients. Taken together, these results indicate that KIF23 is a potential marker for predicting tumor malignancy and prognosis.

In order to explore the biological processes of KIF23 in glioma, KIF23 correlated genes were analyzed by Pearson correlation. We observed a positive correlation of KIF23 expression with a series of tumor oncogenes, driving cell proliferation, mitosis and metastasis, including E2F transcription factor family (E2F1, E2F1, E2F3) [27, 28] and Rhoc [29, 30]. Consequently, KIF23 positive associated genes were analyzed by Gene ontology. The GO terms showed that biological processes of KIF23 associated gene sets were cell cycle process, nuclear division, cell mitosis and chromosome segregation, and the KEGG pathway mainly denoted dysfunction of cell cycle. Additionally, functional assays showed that reduction of KIF23 expression significantly suppressed U87MG cells proliferation in vitro and intracranial tumor growth in vivo, and prolonged intracranial glioma mice's overall survival days by compared to the control group. All these analysis results are in concordance with the reported function that KIF23 is the key regulator of cell cytokinesis.

To further explore the upstream factors from which modulate KIF23 expression, the gene promoter region of KIF23 for regulating DNA-binding elements was searched. Among the entire candidate transcriptionally regulators, we focused on transcription factor 4 (TCF-4), which showed 5 putative binding sites in the KIF23 promoter region. TCF-4 is the core component of the classical Wnt/β-catenin/TCF pathway [20], which interacts with β-catenin in the nucleus to form a complex thereby regulates the transcription of multiple genes to affect cell proliferation, differentiation, survival, and apoptosis. TCF-4 was elevated with increased pathological grade of glioma, and reduction of TCF-4 could suppress glioma growth both in vitro and in vivo, indicating TCF-4 plays an important role in glioma progression and prognosis [31, 32]. In our study, 5 putative TCF-4 binding sites were identified in the promoter region of the KIF23, and the binding site 5 located at −814/−805 bp was validated by ChIP and luciferase reporter assays, implying KIF23 was transcriptionally regulated by TCF-4. To our knowledge, this is the first demonstration that TCF-4 regulates KIF23 expression at transcriptional level.

In conclusion, our study showed that expression levels of KIF23 increasing with the ascending grade of glioma. KIF23 was preferentially expressed in IDH1 wild-type patients, Classical subtype and G3 subtype. It was associated with a worse overall survival and an independent prognostic biomarker in glioma patients. Moreover, silencing KIF23 expression could significantly suppress cell proliferation both in vitro and in vivo. Finally, we demonstrated that KIF23 was regulated by TCF-4 at the transcriptional level. All of results suggested that KIF23 was a novel biomarker and could be a potential therapeutic target for personalized medicine.

## MATERIALS AND METHODS

### KIF23 expression analysis in datasets

Whole genome mRNA expression microarray data and clinical information of 305 glioma samples from CGGA database (http://www.cgga.org.cn) were obtained as the discovery set. The Repository for Molecular Brain Neoplasia Data (REMBRANDT, http://caintegrator-info.nci.nih.gov/REMBRANDT), GSE16011 data (http://www.ncbi.nlm.nih.gov/geo/query/acc.cgi?acc=GSE16011) and The Cancer Genome Atlas (TCGA) RNA sequence database (http://cancergenome.nih.gov) were obtained as validation sets. In the four datasets, only samples with definite WHO classification were included for survival and grade expression pattern analysis. Thus, 305 glioma samples (126 Grade II, 51 Grade III and 128 Grade IV) of CGGA, 688 glioma samples (256 Grade II, 263 Grade III and 169 Grade IV) of TCGA, 317 glioma samples (99 Grade II, 84 Grade III and 134 Grade IV) of REMBRANDT, and 268 glioma samples (24 Grade II, 85 Grade III and 159 Grade IV) of GSE16011 were used for survival and grade expression pattern analysis.

### Gene ontology (GO) analysis of KIF23 associated genes

The Pearson correlation analysis of KIF23 and other genes in whole genome gene expression profile was performed in CGGA dataset. To detect the biological processes that correlate with KIF23 expression in glioma, KIF23 positively correlated genes (R>0.5, P<0.01) were analyzed by DAVID (http://david.abcc.ncifcrf.gov/home.jsp).

### Cell culture and transfection

Cell culture and transfection were done as previously reported [33]. Small interfering KIF23 (si-KIF23) lentivirus and TCF-4 siRNAs were constructed by GenePharma Co. Inc. (Shanghai, China). TCF-4 siRNAs sense sequence is 5′-TTCTCCGAACGTGTCACGT-3′, NC sense sequence is 5′-AGACGAGGGCGAACAGGAG-3′ [31].

### RNA extraction and PCR

Total RNA extraction and PCR assay were performed as previously reported [12]. Primers for KIF23, TCF-4 and GAPDH were as follows, KIF23 forward primer: 5′- CTGACCCAGAGCAAAGCTTTC-3′, reverse primer: 5′- GTTCTAAAGTGCATTCTTGCAGC-3′. TCF-4 forward primer: 5′-CGAGGGTGATGAGAACCTGC-3′; reverse primer: 5′-CCCATGTGATTCGATGCGT-3′; GAPDH forward primer: 5′-CCACCCATGGCAAATTCCATGGCA-3′, reverse primer: 5′-TCTAGACGGCAGGTCAGGTCCACC-3′.

### Chromatin immunoprecipitation (ChIP)

The ChIP assays were performed by using a ChIP assay kit (Upstate Biotechnology) according to the manufacturer's instructions. Briefly, U87 glioma cells were fixed with formaldehyde for 10 min, then lysed in SDS lysis buffer. The chromatin DNA was extracted and sonicated into 200–1000 bp fragments. Immunoprecipitation was performed with 10 μl of the sample used as the “input”, or 1 μg of anti-TCF-4, anti-RNA polymerase II (positive control), negative control IgG. Purified DNA was used for PCR amplification. The primers were designed flanking the putative TCF-4 binding sites in the human KIF23 promoter. Binding site 1: forward: 5′- TCTCACCAATTGATATGAGATAT-3′; reverse: 5′- ATGTACACTCCCACCAGCCTGTA-3′. Binding site 2: forward: 5′- GAATGATTTGCAATATCTGTGAA-3′; reverse: 5′- ACAATGCAGGTAAATATGTAGGA-3′. Binding site 3: forward: 5′-AAGGAGTATGATCTAATAGTAAC-3′; reverse: 5′-ATTAATCTAGCCTTCCCCCACC-3′. Binding site 4: forward: 5′-CTGAGGCACTTTCAGTCTTCTTT-3′; reverse: 5′-AGGAAACGACACCCCAGAGTATG-3′. Binding sites 5: forward: 5′-GAGAAGGGGCTAGAGTTCCATGA-3′; reverse: 5′-CTCTTGCCTTAGATGAACTGATT-3′.

### Luciferase reporter assay

The pGL3-wildtype KIF23-promoter was created by ligation of TCF-4 binding sites located at −814/−805 bp in the KIF23 promoter region into the BglII site of the pGL3 control vector. The pGL3-mutant KIF23-promoter was designed from the wild-type KIF23-promoter reporters by deleting the binding sites (Promega, Madison, USA). Cells of 60%–70% confluence in 24-well plates were cotransfected with luciferase reporter vectors and TCF-4 siRNAs. Luciferase activity was analyzed by the Dual-Luciferase Reporter Assay System with the Renilla luciferase activity as an internal control according to the manufacturer's protocols.

### Intracranial tumor assay

Six-week-old female nude mice (animal center of the Cancer Institute of the Chinese Academy of Medical Science) were used in this assay. U87 cells stably expressing luciferase reporter were transfected with si-KIF23 or NC, and then 1.0 × 10^5^ U87 cells were implanted stereotactically. Tumor volumes were measured by Fluc activity using bioluminescence imaging on days 7, 14, and 21. All the procedures involving animal experiment were performed in accordance to standard guidelines under a protocol approved by Ethics Committee of Capital Medical University.

### Clonogenic and cell growth assays

The Clonogenic andCell growth assays were performed as previous method [11].

### Western blot

Western blot was applied as previous done [33]. Immunoblot analysis was performed with rabbit anti-KIF23 polyclonal antibody (Abcam, 1:800), Rabbit anti-TCF4 polyclonal antibody (Abcam, 1:1000) followed by HRP-conjugated secondary antibodies. Glyceraldehyde-3-phosphate dehydrogenase (GAPDH) was used for control.

### Statistical analysis

Statistical analysis was performed using SPSS Graduate Pack 16.0, R 3.2.1 and GraphPad Prism 5.0 statistical software. Descriptive statistics were shown as mean ± standard deviation. Student's t test, one-way ANOVA test were used to test the significance of differences. Overall survival time (OS) was calculated from the date of histological diagnosis until death or the last follow-up. Kaplan-Meier survival analysis was used to estimate the survival distributions, and the log-rank test was used to assess the statistical significance between stratified survivals groups, patients with lower than median level of KIF23 was defined as low expression, while patients with higher than the median value or equal to the median one was considered as high expression. Univariate and multivariate Cox regression analysis including gender, IDH1 mutation state, tumor grade, age and karnofsky performance status (KPS) were used to assess prognostic value of KIF23 in glioma. A two-sided p value < 0.05 was considered statistically significant.

## SUPPLEMENTARY FIGURES AND TABLES







## Abbreviations

WHO: World Health Origination
IDH1: Isocitrate dehydrogenase 1
MTT: 3-(4, 5-Dimethylthiazol-2-yl)-2, 5-diphenyltetrazolium Bromide
PCR: Polymerase Chain Reaction
ChIP: Chromatin Immunoprecipitation
TCF-4: Transcription factor 4
