# Supplementary material for: KIF23 is an independent prognostic biomarker in glioma, transcriptionally regulated by TCF-4
Source: Oncotarget. 2016 Mar 22;7(17):24646–55. doi: 10.18632/oncotarget.8261 (PMC5029730; doi:10.18632/oncotarget.8261)
Supplement: Supplementary file 1 [file oncotarget-07-24646-s001.pdf]

SUPPLEMENTARY FIGURE AND TABLES

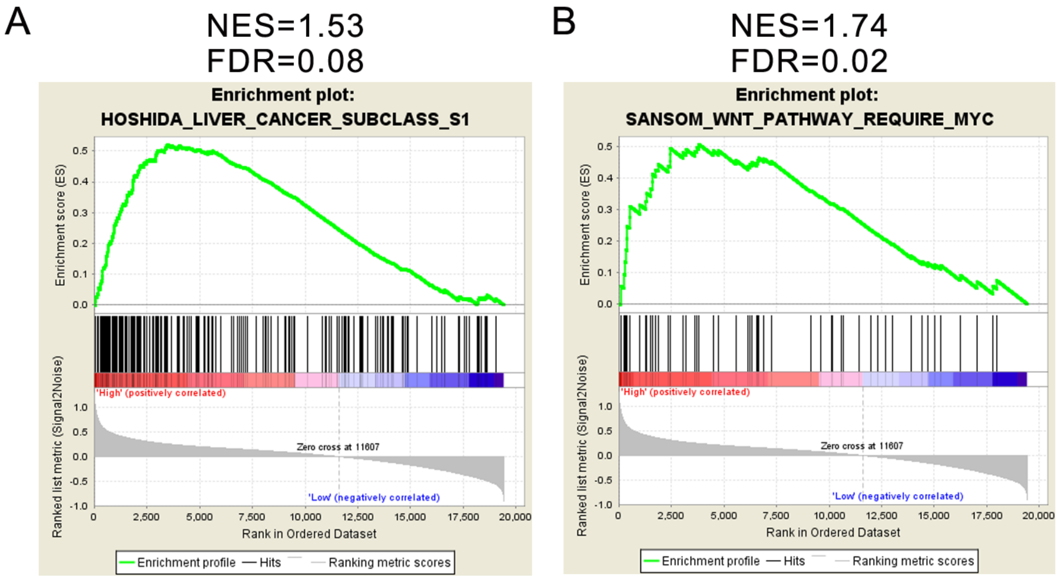

Genes from 'subtype S1' signature of hepatocellular carcinoma (HCC): aberrant activation of the WNT signaling pathway.

Wnt target genes up-regulated after Cre-lox knockout of APC in the small intestine that require functional MYC

Supplementary Figure S1: The expression of KIF23 was tightly associated with wnt pathway activity. GSEA was applied to CGGA microarray dataset with two wnt pathway acticity associated with gene sets.

Supplementary Table S1: KIF23 correlated genes.

See Supplementary File 1

Supplementary Table S2: Cell cycle associated genes of KIF23 correlated genes.

See Supplementary File 2
